# Establishment and characterization of patient-derived xenografts for hormone-naïve and castrate-resistant prostate cancers to improve treatment modality evaluation

**DOI:** 10.18632/aging.102854

**Published:** 2020-02-24

**Authors:** Pengpeng Wu, Rong Xu, Xue Chen, Ya Zhao, Dengxu Tan, Yong Zhao, Weijun Qin, Caiqin Zhang, Xu Ge, Changhong Shi

**Affiliations:** 1 Division of Cancer Biology, Laboratory Animal Center, The Fourth Military Medical University, Xi’an, Shaanxi 710032, China; 2 Biomedicine Application Laboratory, School of Life Science and Technology, Xidian University, Xi’an, Shaanxi 710071, China; 3 Department of Urology, Xijing Hospital, Fourth Military Medical University, Xi’an, Shaanxi 710032, China

**Keywords:** prostate cancer, patient-derived xenografts, androgen, castration, docetaxel

## Abstract

Prostate cancer (PC) is a heterogeneous disease characterized by variable morphological patterns. Thus, establishing a patient-derived xenograft (PDX) model that retains the key features of the primary tumor for each type of PC is important for appropriate evaluation. In this study, we established PDX models of hormone-naïve (D17225) and castration-resistant (B45354) PC by implanting fresh tumor samples, obtained from patients with advanced PC under the renal capsule of immune-compromised mice. Supplementation with exogenous androgens shortened the latent period of tumorigenesis and increased the tumor formation rate. The PDX models exhibited the same major genomic and phenotypic features of the disease in humans and maintained the main pathological features of the primary tumors. Moreover, both PDX models showed different outcomes after castration or docetaxel treatment. The hormone-naïve D17225 PDX model displayed a range of responses from complete tumor regression to overt tumor progression, and the development of castrate-resistant PC was induced after castration. The responses of the two PDX models to androgen deprivation and docetaxel were similar to those observed in patients with advanced PC. These new preclinical PC models will facilitate research on the mechanisms underlying treatment response and resistance.

## INTRODUCTION

Prostate cancer (PC) shows high genetic and phenotypic heterogeneity, which manifests in the classification and histological characteristics of the tumor alongside the growth rate and metastatic capacity between distinct lesions [1, 2]. Androgen deprivation therapy (ADT) is the standard treatment for patients with advanced PC [3]; however, most patients develop castrate-resistant prostate cancer (CRPC) after 2–3 years, which often remains androgen-dependent and is essentially untreatable [4]. Different stages of PC also exhibit considerable disease heterogeneity, requiring distinct treatment methods. Therefore, animal models that mimic the diversity and progression of various types of PC are urgently needed [5, 6]. Moreover, such models are expected to guide treatment decisions for patients with PC.

Conventional cell-line-derived xenograft (CDX) models lack heterogeneity that is associated with tumors, and grow in microenvironments that differ substantially from the conditions in human tumors. PC research is limited by the availability of tissues for molecular studies and human PC cell lines that express intact androgen receptor (AR) and exhibit androgen dependency [7, 8]. One approach to overcome these limitations is through the development of a patient-derived xenograft model (PDX)—which involves the direct implantation of fresh cancer tissue specimens into immunodeficient mice—a clinically relevant model that can accurately capture the "omic" diversity of PC [9–11].

The efficacy of a PDX model has been reported to be similar to that in clinical patients. Most studies demonstrated that the PDX model could be used to screen therapeutic drugs in mice, thereby providing a reasonable treatment strategy [12, 13]. Therefore, diverse PC PDX models are urgently needed to simulate the development of PC—from primary tumor to metastasis, and from androgen-dependent to androgen-independent disease—in mice so that this model reflects the diversity observed in patients. The ideal PC PDX model will partly simulate the clinical transformation process and the mechanism of transition of tumors from hormone-naïve prostate cancer (HNPC) to CRPC, and from adenocarcinoma to neuroendocrine carcinoma [14–16].

To date, aside from PDX models from hormone naïve primary prostate cancer, representative PC PDX models include the LAPC, KUCaP, and LuCaP series [17, 18]. These were all derived from patients with advanced-stage PC and represent only a small proportion of phenotypes. Thus, the current models do not fully recapitulate the disease heterogeneity. Application of the results obtained from PDX models in a clinical setting has been limited by the long incubation period, hormone dependence, and low success rate of the graft that closely mimics the conditions, characteristics, and treatment response of clinical patients [19]. Therefore, it is necessary to establish PC PDX models with various clinical pathological characteristics that can mimic the full stage of disease progression and represent patient populations with different backgrounds. In this study, we established PDX models of HNPC and CRPC, evaluated their major genomic and phenotypic features, and assessed their response to androgen deprivation and docetaxel treatments. These models can be used to expand the knowledge of the biology of PC and guide more personalized treatment decisions.

## RESULTS

### Characteristics of the PDX models

**Figure 1:**
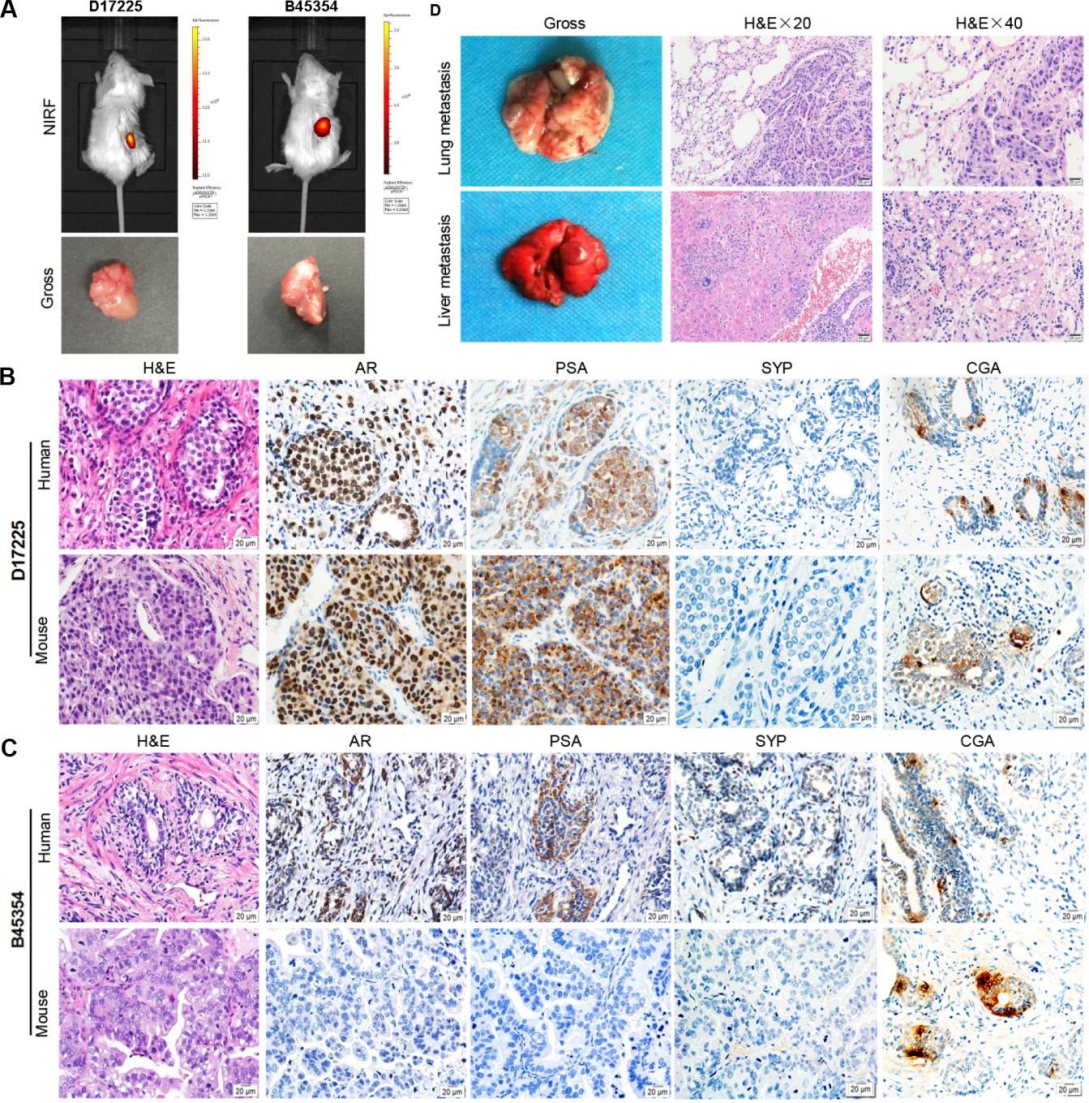
**Establishment of molecular heterogeneous PDX models with maintained pathological features of patient tumors.** (**A**) NIRF optical imaging of NPG mice with PC xenograft tumors using MHI-148 dye (Top). The PDX models of PC were established by implantation of clinical tumor specimens D17225 and B45353 into the renal capsule of NPG mice. Gross morphology of PC tissue-implanted mouse kidney (bottom). (**B**, **C**) H&E and IHC analyses of tumor tissues derived from both PDX models and patient samples (D17225 and B45353). (**D**) Multiple organ metastasis PDX models from B45354 (CRPC patient). H&E staining of metastatic tumors (including the lung and liver). Original magnification, 400×; scale bars represent 20 μm.

Fresh surgical PC specimens, from a patient with CRPC (B45354) and from another with HNPC (D17225) were used to establish two PC PDX models with differing androgen sensitivities. The tumor specimens were implanted into the renal capsule of male immune-compromised NPG/Vst (NOD-*Prkdc* scid *Il2rg*null) mice and supplemented with testosterone. Initial tumor growth was measured with a near-infrared fluorescence (NIRF) optical imaging system twice weekly using the NIRF heptamethine carbocyanine dye MHI-148. The dye was seen to have preferential uptake in tumor cells but not in normal cells. This can be employed directly for non-invasive dual imaging and for the targeting of agents in human cancers without the need of chemical conjugation [20, 21]. As shown in Figure 1A (top), NIRF signals with strong intensity were obtained in the kidney 3 months after the transplantation of D17225 or B453543 specimens, indicating tumor formation. After euthanizing the mice, tumor nodules were observed (Figure 1A, bottom). Tumors that grew were dissected from the host mice and further passaged subcutaneously into new NPG/Vst male mice. The initial PDX model was established within 3–6 months. We considered three passages to indicate of an established PDX line.

### Fidelity of the PDX models to the primary tumor

Both the D17225 and B45354 PDX models exhibited diverse histological features (Table 1). Hematoxylin and eosin (H&E) staining confirmed that the PDX tumor tissues exhibited identical morphological characteristics to that of the original tumor. In order to distinguish between human and mice tissues, the human mitochondria was used as a marker, and was strongly expressed in both human xenograft tissues (Supplementary Figure 1). In the D17225 PDX, the tumor basal cells disappeared and a large number of irregular sieve-like structures appeared. The cancer cells were enlarged and infiltrated the surrounding space. Castration-resistant B45354 PDX tumors retained the glandular structure of the primary tumor with relatively clear boundaries. In the D172225 PDX, both AR- and prostate-specific antigen (PSA)-specific immunohistochemistry (IHC) profiles were generally maintained (Figure 1B and 1C). Additionally, the expression levels of the neuroendocrine carcinoma markers synaptophysin (SYP) and chromogranin A (CGA) were concordant with those in the original tissue. However, the expression of most of the proteins in the B45354 patient were only weakly positive, whereas that in the B45354 PDX mice were negative; only CGA was weakly expressed in the PDX model. Moreover, high metastatic potential was observed in the B45354 PDX model, partly because of greater vascularization at the graft site, and multi-organ metastases ultimately developed, including in the lung and liver (Figure 1D). The tumor cells invaded the right seminal vesicles and bladder tissue, resulting in a higher degree of malignancy than that observed in the patient. The tumor of castration-resistant B45354 reached a maximum volume of 800 mm^3^ 4 weeks after implantation and grew faster than the androgen-dependent D17225 tumor.

**Table 1:** Characteristics of patients’ tumor and matched PDX tumor.

**ID**	**Pathology**	**Hormone**	**Chem**	**STR**	**PSA**	**AR**	**CGA**	**SYP**
D17225	Ad	De	No	NA	+	+	±	-
D17225 -PDX	Ad	De	——	human	+	+	±	-
B45354	Ad	Re	No	NA	+	+	±	±
B45354- PDX	Ad	Re	——	human	-	-	±	-

Note: PSA: Prostate Specific Antigen; AR: Androgen Receptor; CGA: Chromograin A; SYP: Synaptophysin; NA: Not Available; Ad: Adenocarcinoma; De: depend; Re: resistance; Chem: Chemotherapy.

### Factors influencing establishment of the PC PDX models

**Figure 2:**
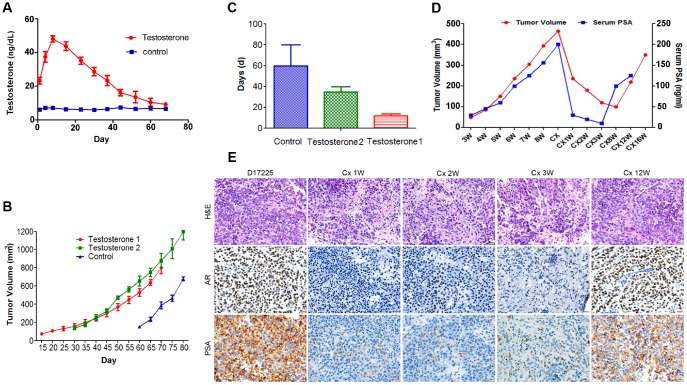
**Characteristics of the hormone-naïve D17225 PDX model.** (**A**–**C**) Factors influencing the establishment of the PDX model. (**A**) Change in testosterone levels in androgen-implanted mice. (**B**) Effect of supplementation with androgen on PC tumor growth. (**C**) Effect of supplementation with androgen on the growth latency of the PC tumor. Results are shown as the means ± SD (n = 5). (**D**–**E**) Induction of CRPC in the D17225 PDX model. (**D**) Tumor volumes of D17225 xenografts and mouse serum PSA levels at various time points before, during, and after castration-induced CRPC development. (**E**) H&E staining of D17225 tumor sections, and the levels of AR and PSA in tumor sections (IHC analysis) at various time points before and after host castration and in recurrent tumors. The scale bar indicates 100 mm. Cx indicates castration weeks.

To examine the main factors influencing the establishment of the PC PDX models, tumor tissue from the D17225 PDX model was implanted into mice. The control group was only subcutaneously implanted with the tissue, whereas in the testosterone groups implantation was performed subcutaneously (testosterone 1) or under the renal capsule (testosterone 2) and testosterone was supplemented. The serum testosterone levels in androgen-implanted mice were measured weekly. After one week, the testosterone levels reached a peak and then gradually decreased but remained between 20–60 ng/dl (Figure 2A), significantly higher than that of normal male mice (6.0-7.8 ng/dl). Mice supplemented with androgen displayed faster tumor growth than those in the control group (Figure 2B). The renal capsule transplantation group exhibited significantly shortened growth latency (from the first day of transplantation to the time of tumor growth to 100 mm^3^) (Figure 2C).

### Induction of CRPC

One week after the castration of mice bearing D17225 tumors, the tumor volume was markedly reduced, accompanied by a substantial decrease in host serum PSA levels (Figure 2D). This mimics the clinical response of patients with PC to ADT. A few months after castration, the host serum PSA levels increased (Figure 2D). One week after host castration, tumor growth slowed, and AR expression markedly decreased, indicating reduced AR transcriptional activity. However, after tumor recurrence, these markers were positively expressed at higher levels (Figure 2E).

### Short tandem repeat (STR) profile and AR mutation

**Figure 3:**
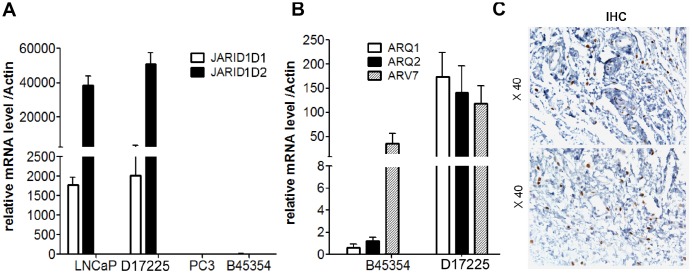
**Expression of *AR* and *JARID1D* by RT-PCR analysis.** (**A**) Expression of *JARID1D* in tumors tissue of different PDX models and PC cells were detected by RT-PCR. (**B**) Expression of *AR* and *ARV7* in tumors tissues of different PDX models detected by RT-PCR. Data are shown as the means ± SD of three independent experiments performed in triplicate. (**C**) Expression of *ARV7* in tumors tissues of B45354 PDX models detected by IHC. Original magnification, 400×; scale bars represent 20 μm.

To ensure the originality and maintenance of the genotypes of the PDX models, we performed STR analyses, and demonstrated distinct STRs in each model. The 16 STR loci of tumor tissues derived from PDX models were confirmed to be identical to the loci from the patient tumor tissue (Table 2). Further analysis demonstrated the loss of *JARID1D* expression in the tumor tissue from the B45354 PDX, which was similar to the pattern observed in the PC cell line, PC-3 (Figure 3A). This gene—located on the Y chromosome—is a prognostic marker and suppressor of prostatic tumor invasion and metastasis [22, 23]. Polymerase chain reaction (PCR) from B45354 barely amplified the specific *AR* band, whereas D17225 showed an amplified band and the fragment size was consistent with that of the *AR* target fragment after sequencing. The expression of *AR*, as determined by reverse transcription (RT)-PCR analysis was consistent with the results of IHC. In addition, although *AR* expression was nearly negative in B45354 tumor tissues, an increase in the levels of the androgen receptor variant 7 (*ARV7*) was detected in the PDX tissue by RT-PCR, but weak positive expression was detected by IHC (Figure 3B and 3C).

**Table 2:** STR analysis of PDX models.

**Locus**	**Range of Alleles**	**D17225**	**B45354**
Amelogenin	-	X,Y	X
D8S1179	7-20	14, 15	13, 16
D21S11	12-41.2	29, 32.2	32.2
D7S820	5-16	8, 10	10, 11
CSF1PO	5-16	12, 14, 15	12
D3S1358	8-21	14, 16	15, 18
D5S818	7-18	11, 14	10
D13S317	5-16	8, 10	8, 13
D16S539	5-16	9, 12	9, 12
D2S1338	15-28	20, 24	19
D19S433	9-17.2	13, 15.2	13
D12S391	8-28	16, 19	17, 18
D18S51	7-39.2	14, 16	15
D6S1043	7.1-23.3	13	11
vWA	10-25	14, 15	16, 18
FGA	12.2-51.2	22, 30	22, 26

### Responses to castration and docetaxel

**Figure 4:**
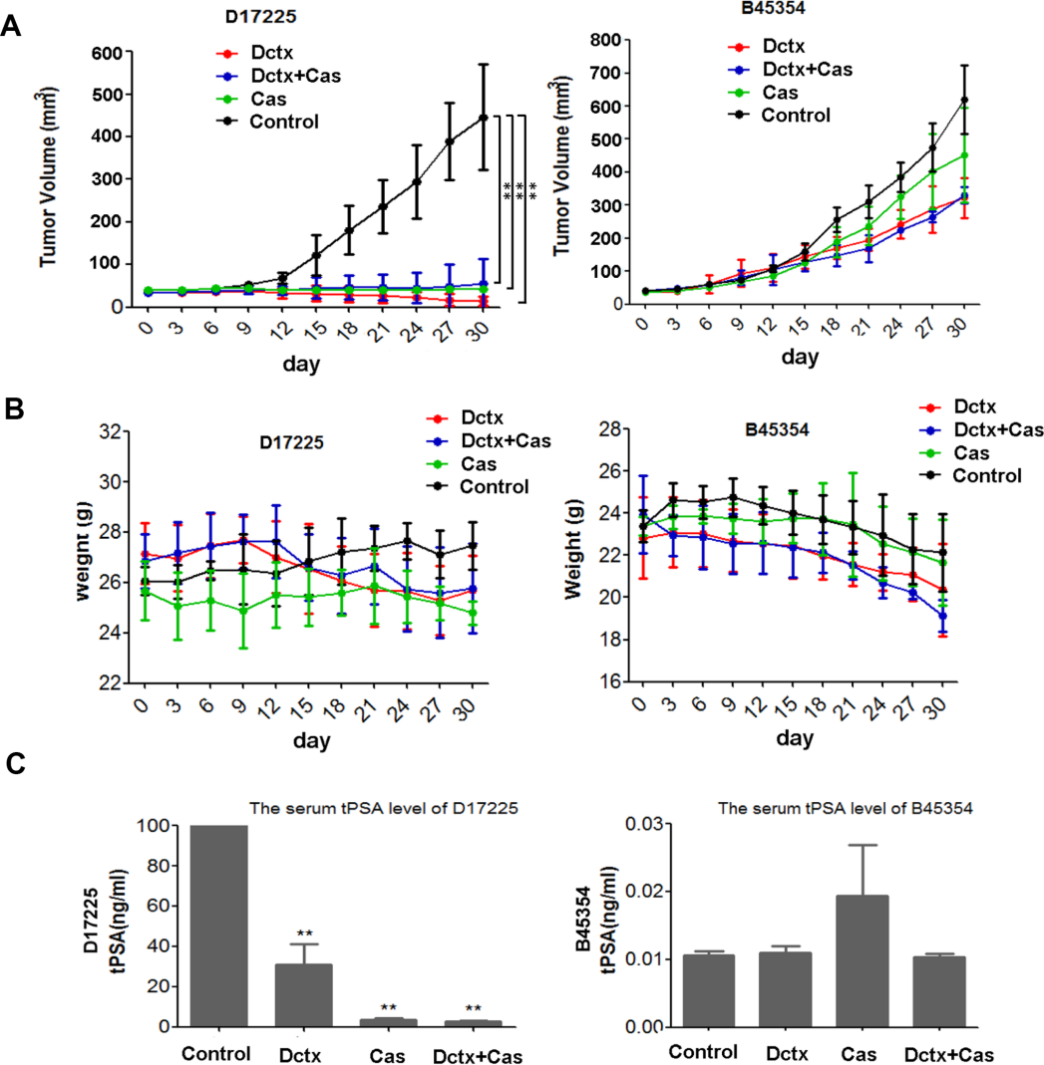
**Responses to castration and docetaxel in PDX models.** Change in the (**A**) tumor volume and (**B**) weight of PDX models after treatment at various time points. (**C**) Serum tPSA levels in mice bearing PDX tumors after treatment for 30 days. **P < 0.01 compared to the control.

Because ADT is the first-line therapy for advanced PC and docetaxel was the first therapeutic agent shown to extend survival in men with metastatic CRPC [3], we evaluated the responses of different PDX models to castration, docetaxel and combination of the two. The therapeutic effect was assessed by examining changes in the tumor volume and body weight of tumor-bearing mice. As shown in Figure 4A, in the D17225 PDX model, docetaxel (Dctx), Castration (Cas) or the combined castration + docetaxel (Dctx+Cas) treatment significantly inhibited tumor growth *(P* < 0.01 compared to controls). In the B45354 PDX model, although docetaxel also inhibited tumor growth, there was no significant difference with respect to the control group, and both castration + docetaxel and docetaxel treatments resulted in significant loss in the body weight of the mice (Figure 4B).

The serum total prostate specific antigen (tPSA) level is an important indicator used in the clinical diagnosis and treatment evaluation of PC [1]. In the D17225 model, tPSA levels in all tumor-bearing mice were above 100 ng/mL. The tPSA levels were the lowest in the docetaxel + castration group (average 2.58 ng/mL), followed by the castration group (average, 3.65 ng/mL). In contrast, the average tPSA content in the docetaxel group was 30.92 ng/mL (Figure 4C). In the B45354 model, the serum tPSA level was below the critical minimum in all mice, which was consistent with the results of histopathological analysis.

**Figure 5:**
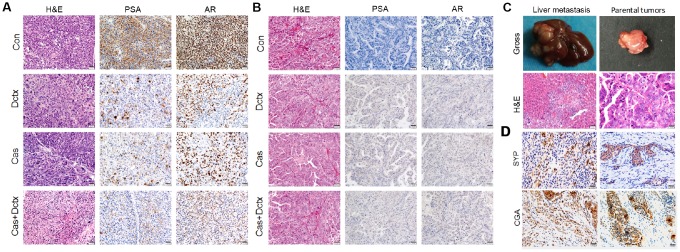
**H&E staining and IHC analysis of different treatment groups.** Results of H&E staining and IHC analysis in the different treatment groups in the (**A**) D17225 and (**B**) B45354 PDX model. (**C**) Representative images of liver metastasis and the parental tumors after treatment with docetaxel in the B45354 PDX model. (**D**) Expression of neuroendocrine carcinoma markers SYP and CGA after treatment with castration + docetaxel in the B45354 PDX model.

We also evaluated the therapeutic effect by observing the changes in histological morphology with H&E staining (Figure 5). In the D17225 model, the tumor cells were abundant and varied in size, and the morphology of the nucleus was variable. After castration or docetaxel treatment, the number of tumor cells decreased and the matrix component increased. After castration + docetaxel treatment, the number of tumor cells decreased gradually and did not exhibit typical characteristics (Figure 5A). In the B45354 model, the different treatments did not alter the structure of the tumor tissue significantly (Figure 5B). The model even displayed liver metastasis after treatment with docetaxel (Figure 5C) with metastatic percentage reaching 30%. Moreover, the treatment of B45354 PDXs with castration + docetaxel induced the expression of the neuroendocrine markers CGA and CYP at high levels, thus displaying a feature of neuroendocrine carcinoma (Figure 5D).

## DISCUSSION

In establishment of a PC PDX model, the PC tissues extracted from patients presumably contain a mixture of growth-arrested, androgen-dependent tumor cells, as well as androgen-independent cells at the time of implantation into mice [24, 25]. In intact male mice, androgen-dependent cells have a growth advantage and eventually develop into androgen-sensitive xenografts. Some PDX models also exhibit castration resistance after castration. Thus, these models accurately mimic the main clinical types of PC characterized by hormone-dependence and castration-resistance [1, 26]. In this study, we selected two types of PC surgical specimens from patients to establish the corresponding PDX models. The clinical histopathology of PC is mainly characterized by basal cell destruction or disappearance, gland abnormalities, nuclear atypia, and infiltration [27, 28]. Both PDX models maintained the structural characteristics of the primary tumor from the corresponding patients. Two well-established PC markers, PSA and AR, were found to maintain the same expression level in D17225 PDX tumors that in the primary tumor. However, in D45354 PDX tumors the expression of PSA and AR decreased, which may be due to individual differences in patients and species differences between humans and mice. Correspondingly, the PSA level in the serum of the B45354 patient remained low (only 19.18 ng/mL). The consistency of the hormone-dependency status of the primary tumor was further confirmed by surgical castration.

To improve the success rate of PC PDX model development, we applied a series of strategies. The level of androgen in mice is much lower than that in adult men, and a certain dose of androgen stimulation is essential for the growth of tumor tissues [29]. Therefore, we supplied the host mice with exogenous androgen (androsten-17β-01-3-one) to promote tumor growth. Significant high level of testosterone was induced by supplemented androgen, but it did not reach the average level in males, which may be due to species differences between humans and mice. In addition, we optimized the transplant site. The model establishment rate obtained by conventional subcutaneous transplantation is only approximately 10% [30]. The subrenal capsule, which has a rich vascular structure, provides sufficient nutrients, hormones, and oxygen to promote early tumor tissue growth, and is considered to be a suitable site for PC tissue xenografts [31]. Further, the tumor tissue was maintained in a fresh state. Patient specimens obtained during surgery should be accurately trimmed and transplanted into mice as soon as possible to reduce the incidence of tissue liquefaction and necrosis [32].

Neuroendocrine prostate cancer (NEPC), an aggressive variant of PC, is often observed in patients with progressive CRPC after treatment with drugs that target AR signaling [33]. However, the mechanisms contributing to the progression to prostatic NEPC remain unclear [34]. In this study, D45354, as a typical adenocarcinoma, expressed low levels of AR and PSA, but further treatment with castration + docetaxel induced the expression of the neuroendocrine markers CGA and CYP, indicating that this model had the characteristics of neuroendocrine carcinoma. The current lack of understanding of the genetic and epigenetic mechanisms underlying NEPC makes diagnosis difficult, and has further hindered the development of treatments against this aggressive disease [35]. Therefore, the D45354 PDX model offers a potential tool for developing effective therapies and evaluating tumor responses to targeted drugs for aggressive NEPC. Based on our findings, we can hypothesize that post-treatment NEPC develops clonally from an adenocarcinoma precursor.

ARV7, the most common variant of AR, plays an important role in the clinical treatment of drug resistance, and its expression is associated with the malignancy and metastasis of PC, which affects treatment outcome and prognosis [36]. Although some patients with PC express low level androgens, ARV7 can continuously activate the AR signaling pathway, leading to resistance to multiple treatments while promoting disease progression and metastasis [37]. In this study, the expression of *ARV7* mRNA was detected in both PDX models. In the B45354 model, although IHC showed negative expression of the AR protein, the levels of *ARV7* remained at a certain level, indicating the development of drug resistance and a malignant phenotype. It is difficult to verify such low levels of expression having any biological significance. It implies that the activation of *ARV7* signaling in B45354 requires further experimental verification. The B45354 PDX model developed multiple organ metastases (including to the liver and lung) during passaging in mice, showing a high degree of malignancy and a poor therapeutic effect. Nguyen et al. [3] found that *ARV7* expression was markedly upregulated in their LuCaP PDX model of CRPC, suggesting that this model can be used in the development of novel AR N-terminal antagonists. Therefore, targeting *ARV7* has emerged as a new treatment strategy for PCs, particularly for patients with relapsed or resistant PC.

Loss of the Y chromosome in the peripheral blood was found to be correlated with the high risk of cancer [38]. The entire or partial regions of the male-specific Y chromosome are deleted in up to 52% of prostate tumors. The male-specific JARID1D gene encodes a histone H3 lysine 4 demethylase, and is highly downregulated in metastatic prostate tumors relative to that in normal prostate tissues and primary prostate tumors [39]. Furthermore, *JARID1D* is frequently deleted in metastatic prostate tumors, and its low levels are associated with a poor prognosis in patients with PC [23]. Consistently, *JARID1D* was barely amplified in the B45354 PDX model with features of multi-organ metastasis, thereby confirming that it is an effective metastatic marker of PC.

Tumors from the hormone-dependent D17225 PDX model exhibited markedly reduced volumes after castration surgery or docetaxel treatment, whereas no apparent effect of these treatments was observed on the castration-resistant B45354 tumors. Body weight continuously decreased and liver metastasis was observed in the host mice, potentially due to the toxic side effects of the chemotherapeutic drugs or due to the high degree of malignancy of the B45354 tumor. In this study, the drug treatment was stopped when the body weight decreased by 20%. This significant decrease in body weight suggests that the follow-up treatment of patients should be appropriate. Weight loss is not only a side effect of docetaxel, but is also a consequence of specific interactions among the tumor, chemotherapy, and the host.

The development of CRPC is currently the major challenge in the management of advanced PC. It is partly hindered by the lack of clinically relevant cancer models, especially models based on patient-derived HNPCs that develop CRPC following host castration [11]. The D17225 PDX model established in this study effectively mimicked the patient’s treatment responses, as evidenced by CRPC development after castration, offering a better understanding of PC with the aim of improving the outcome.

In summary, we successfully established two PC PDX models with distinct clinical features, and confirmed their similarities to the primary tumors of the respective patients. Castration or docetaxel treatment inhibited the growth of HNPC D17225 tumors, but had no apparent effect on the CRPC B45354 tumors. We further induced the HNPC PDX models to develop CRPC by castration. The responses of the two PDX models during treatment were similar to those of the clinical patients, providing reliable data to support individualized treatments such as evaluation of treatment efficacy and prognosis.

## MATERIALS AND METHODS

### Animals

Six to seven-week-old male NPG/Vst (NOD-*Prkdc* scid *Il2rg* null) mice were purchased from Beijing Vitalstar Biotechnology (Beijing, China) and housed in a pathogen-free environment at the Laboratory Animal Center of the Fourth Military Medical University (FMMU, Xi’an, China). The mice were anesthetized with ketamine (100 mg/mL, i.p.) and xylazine (20 mg/mL, i.p.) and maintained under isoflurane during surgery and imaging. All animal experiments were approved by the Institutional Animal Care and Use Committee of FMMU (Protocol No. 16013).

### Cells and tumor specimens

Both human prostate cancer cell lines LNCaP and PC3 were purchased from the American Type Culture Collection (ATCC, Manassas, VA). Fresh prostate cancer specimens were obtained from the Xijing Hospital of the FMMU, and informed consent was obtained from both patients. One specimen was derived from a clinical HNPC patient (D17225) and the other was derived from a CRPC patient (B45354). The use of these specimens in research was approved by the Medical Eethics Committee of Xijing Hospital (KY20193035). After dissection, the specimens were cut into 1–3-mm^3^ fragments and the stromal capsules and necrotic tissue were removed.

### Establishment of the PDX models

Two to three PC specimen grafts mixed with an appropriate amount of matrigel (BD Biosciences, Franklin Lakes, NJ, USA) were implanted between the capsule and underlying parenchyma of each kidney in the male mice under general anesthesia [31]. To maintain host testosterone levels, implants of testosterone (4 Androsten 17b-01–3-one, Sigma Chemical Co., St. Louis, MO, USA) were inserted intraperitoneally at a dose of 40 mg/capsule per mouse [6]. Plasma testosterone levels in tumor-bearing mice with implants were monitored by enzyme-linked immunosorbent assay (ELISA). Initial tumor growth was measured with an NIRF optical imaging system (Caliper Life Sciences, Hopkinton, MA, USA) twice weekly. The mice were intravenously injected with MHI-148 (1 μmol/kg; provided by Dr. Leland W.K. Chung, Cedars-Sinai Medical Center, Los Angeles, CA, USA), and the NIRF intensity in the regions of interest were measured using a Lumina II Imaging System at 24 h after dye administration. The mice were monitored for up to 6 months post-implantation to examine initial tumor growth [20], and MHI-148 uptake was examined in accordance with the published methods [19]. Tumor samples from PDX models were collected at each stage and frozen or fixed with 4% polyformaldehyde.

### STR analysis and PCR amplification

Genomic DNA was extracted from PDX tissues for STR analysis with a tissue genomic DNA extraction kit from Tiangen Biochemical Technology Co., Ltd. (Beijing, China). To evaluate gene expression, total RNA isolated from the PDX tissues was used for RT-PCR as previously described [3, 23]. The primer sequences of the target genes (*AR, AR7, JARID1D*) are listed in Supplementary Table 1.

### Treatment

Male NPG mice were subcutaneously implanted with tumor grafts. When the tumor size exceeded 80–100 mm^3^, the mice were divided into the following four groups: 1) castration group with surgical removal of the bilateral testes and their attachments, 2) docetaxel group that received docetaxel treatment at a dose of 2 mg/kg via tail vein injection twice weekly, 3) castration + docetaxel group, and 4) control group containing non-castrated male mice. The docetaxel injection solution was procured from Sanofi Pharmaceutical Co., Ltd. (Hangzhou, China). Phosphate-buffered saline (control group) was injected via the tail vein twice weekly. The tumor volume and body weight of the mice in the PDX model were measured once every 3 days. The body weight of tumor-bearing mice and the maximum length (l) and width (w) of the tumor were measured; tumor volume (V) was calculated using the formula V = ½ × (w^2^ × l). After 30 days of treatment, the experiment was terminated, and the blood was collected from the mice to determine the tPSA levels in the serum by ELISA. Then, all tumor-bearing mice were euthanized.

### H&E and IHC staining

A section of each tumor was fixed in 4% paraformaldehyde blocks and the morphology was compared to that of the original patient tumor by H&E staining and IHC analysis. The following primary antibodies were used: CGA (1:100; Abcam, Cambridge, UK), SYP (1:50; Santa Cruz), rabbit anti-human anti-PSA, anti-AR monoclonal antibodies (1:100, Abcam, Cambridge, UK). Anti-AR(AR-V7 specific) antibodies (1:200, Abcam, Cambridge, UK) and anti-human mitochondria antibodies (1:100, Abcam, Cambridge, UK). The secondary antibody was a biotin-labeled goat anti-rabbit, which was used according to a published protocol [1].

### Statistical analysis

All data are presented as the mean ± SD from at least three independent experiments. The statistical significance of all data was analyzed by a two-tailed unpaired Student’s *t* test, and a *P* value < 0.05 was considered statistically significant.

## Supplementary Material

Supplementary Figure 1

Supplementary Table 1
