# Supplementary material for: Establishment and characterization of patient-derived xenografts for hormone-naïve and castrate-resistant prostate cancers to improve treatment modality evaluation
Source: Aging (Albany NY). 2020 Feb 24;12(4):3848–61. doi: 10.18632/aging.102854 (PMC7066917; doi:10.18632/aging.102854)
Supplement: Supplementary Figure 1 [file aging-12-102854-s002..pdf]

SUPPLEMENTARY FIGURE

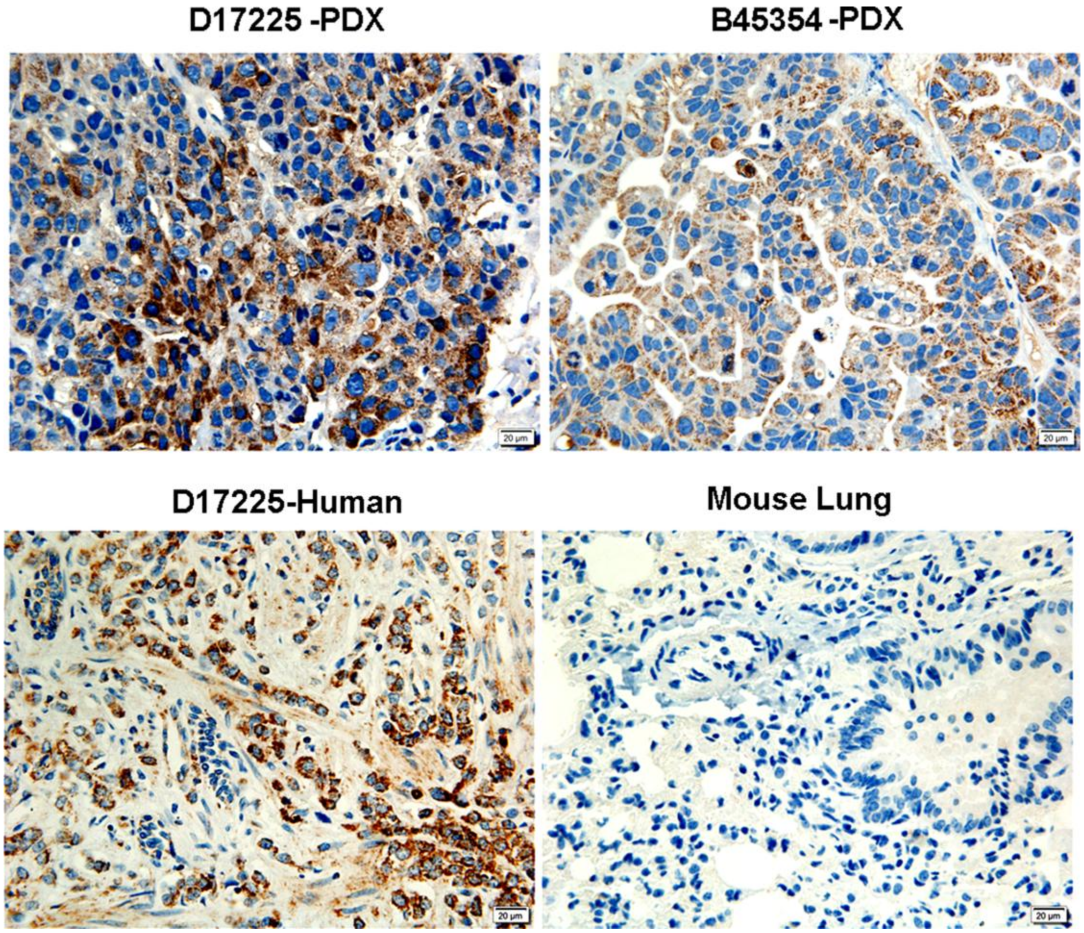

Supplementary Figure 1. The expression of human mitochondria in PDX tissue. Strong positive expression of human mitochondria was detected in both B45354 and D17225 PDX tissues by IHC. Negative expression was observed in normal mouse lung tissue. Original magnification, 200×; scale bars represent 20 μm.
