# Supplementary material for: Establishment and characterization of patient-derived xenografts for hormone-naïve and castrate-resistant prostate cancers to improve treatment modality evaluation
Source: Aging (Albany NY). 2020 Feb 24;12(4):3848–61. doi: 10.18632/aging.102854 (PMC7066917; doi:10.18632/aging.102854)
Supplement: Supplementary Table 1 [file aging-12-102854-s001..pdf]

SUPPLEMENTARY TABLE

Supplementary Table 1. Primer sequences of different gene used for qRT-PCR.

AR	AR1	F: CCAGGGACCATGTTTTGCC
		R: CGAAGACGACAAGATGGACAA
	AR2	F: TCCATCTTGTCGTCTTCGGAA
		R: GGGCTGGTTGTTGTCGTGT
ARV7	ARV7	F:CCATCTTGTCGTCTTCGGAAATGTTATGAA
		R: TTTGAATGAGGCAAGTCAGCCTTTCT
JARID1D	JARID1D1	F: AGCCAACCATGTGCAATGTA
		R: GGCTCTGGATCAGGCTGTAG
	JARID1D2	F: CCAGAGCCTACAGAGGAGGACAT
		R: CCCACCTCCACTTTGCTCAT
